# Investigating the potential of oncolytic viruses for cancer treatment via MSC delivery

**DOI:** 10.1186/s12964-023-01232-y

**Published:** 2023-09-04

**Authors:** Hadi Esmaeili Gouvarchin Ghaleh, Gazal Vakilzadeh, Ali Zahiri, Mahdieh Farzanehpour

**Affiliations:** 1 Applied Virology Research Center, Baqiyatallah University of Medical sciences, Tehran, Iran; 2 Students Research Committee, Baqiyatallah University of Medical Sciences, Tehran, Iran

**Keywords:** Mesenchymal stem cells, Oncolytic viruses, Tumor microenvironment, Immunotherapy

## Abstract

Mesenchymal stem cells (MSCs) have attracted considerable interest as a promising approach for cancer treatment due to their ability to undergo tumor-trophic migration. MSCs possess the unique ability to selectively migrate to tumors, making them an excellent candidate for targeted delivery of oncolytic viruses (OVs) to treat isolated tumors and metastatic malignancies. OVs have attracted attention as a potential treatment for cancer due to their ability to selectively infect and destroy tumor cells while sparing normal cells. In addition, OVs can induce immunogenic cell death and contain curative transgenes in their genome, making them an attractive candidate for cancer treatment in combination with immunotherapies. In combination with MSCs, OVs can modulate the tumor microenvironment and trigger anti-tumor immune responses, making MSC-releasing OVs a promising approach for cancer treatment. This study reviews researches on the use of MSC-released OVs as a novel method for treating cancer.

**Graphical Abstract:**

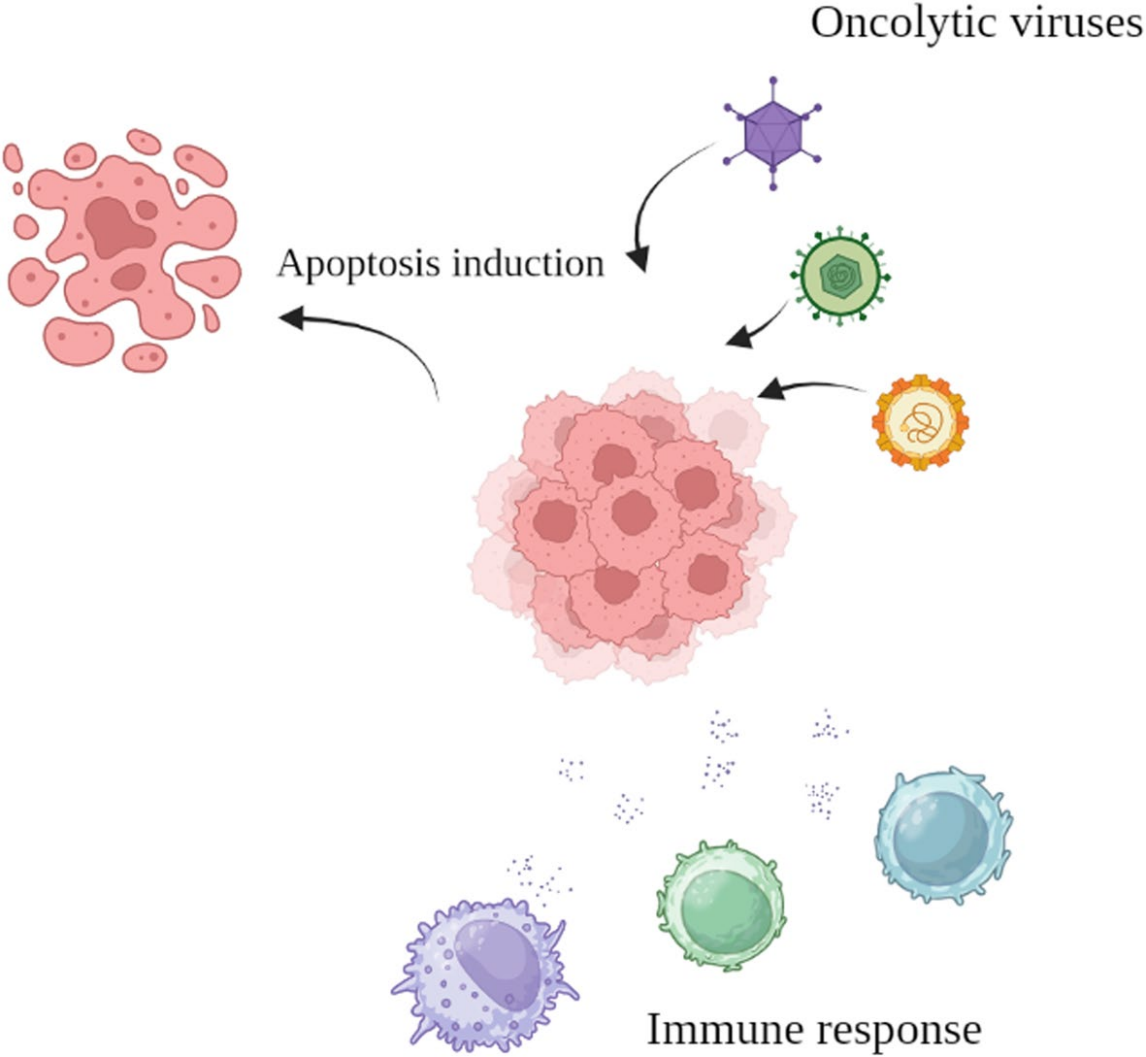

Video Abstract

**Supplementary Information:**

The online version contains supplementary material available at 10.1186/s12964-023-01232-y.

## Introduction

 Cancer therapy has witnessed significant advancements in recent years, leading to improved prognosis for cancer patients. Timely detection of specific cancer types and the development of targeted therapies have played a crucial role in this progress [1, 2]. However, certain limitations such as the short half-life of cancer-specific drugs, limited distribution to specific tumors, and adverse effects on healthy tissues have posed significant challenges to the effectiveness of these treatments [3–6]. Hence, there is a pressing need to design anticancer drugs that can precisely target cancerous cells while minimizing damage to normal tissues [7]. Furthermore, despite the decline in cancer mortality rates reported by the American Cancer Society from 2017 to 2018, achieved through advances in early detection and treatment [1], there remains a demand for innovative approaches to further improve cancer treatment outcomes.

In recent years, several new targeted therapies have been approved by the U.S. Food and Drug Administration (FDA) for the treatment of various types of cancer, exemplified by lung, breast, and bladder cancer [2–4]. These approvals underscore the ongoing efforts to enhance cancer treatment approaches. However, considering the limitations of existing modalities, such as systemic adverse effects and drug resistance, the development of innovative therapeutic approaches that prevent the spread of cancer is crucial [8, 9].

Viruses have gained attention not only for their potential to trigger oncogenesis but also for their application in immunotherapeutic approaches and the advancement of adoptive T-cell immunotherapies targeting cytomegalovirus. In this context, viruses can serve as therapeutic agents for attacking tumor cells [10, 11]. Oncolytic viruses (OVs) refer to viruses that occur naturally or are genetically altered to replicate specifically within cancer cells and cause their destruction, while sparing normal cells [12]. OV therapy, as a novel approach to cancer treatment, has shown promise in preclinical and clinical studies [13]. A variety of viruses, including reovirus, Newcastle disease virus, adenovirus, measles virus, vesicular stomatitis virus, herpes simplex virus, and vaccinia virus, have been used for this therapy [14, 15]. In addition to inducing direct lysis of cancer cells, OVs are known to activate or reactivate cytotoxic immune responses in patients, leading to therapeutic responses [16]. However, the effectiveness of OVs in reaching cancerous tissues is influenced by various factors, including immune system elimination processes and the uptake of viruses by tissues and organs [17]. Therefore, it becomes crucial to enhance treatment efficacy by utilizing effective delivery vehicles for OVs to reach tumor sites [18].

Mesenchymal stem cells (MSCs) have demonstrated potential in the treatment of various diseases, including cancer, owing to their antitumor substances that inhibit the proliferation of cancer cells. Moreover, due to their tumor-homing capabilities, MSCs have been employed as carriers, facilitating safe transport and release of the virus at the tumor site [19, 20]. The use of MSCs as delivery vehicles for OVs offers the potential to increase the amount of OV administered to patients, thereby minimizing side effects and obviating the need for direct injection into the tumor [21]. Therefore, the primary objective of this review is to investigate the potential of OVs delivered via MSCs as a novel approach to cancer therapy. This review aims to highlight the advantages and limitations of using MSCs as delivery vehicles for OVs, while also providing an overview of their effects on tumors.

### Utilizing MSCs as a vehicle for OV delivery

#### I. Tumor-homing ability of MSCs

In the 1970s, a new type of stem cells with the potential to generate multiple lineages was discovered in the marrow stroma. These cells, called colony-forming unit fibroblasts, were fibroblast-like cells that were able to produce colonies in vitro and differentiate into a range of mesenchymal and non-mesenchymal cell types in both in vitro and in vivo settings [22]. During the late 1980s, additional research was conducted to investigate the heterogeneity of the marrow stromal cell population. This led to the discovery of a stem cell subset that belongs to the mesodermal germ layer lineage and is involved in the development of connective tissue, skeletal muscle cells, and the vascular system. These stem cells, identified as bone marrow-derived MSCs (BM-MSCs), have been found to play a crucial role in these physiological processes [23, 24]. The distinctive immunomodulatory characteristics of BM-MSCs in allogeneic transplantation have piqued significant interest [25].

#### II. Protection of OVs from clearance

The use of OVs as a systemic treatment for cancer faces several obstacles that hinder their successful implementation. For example, after administration into the systemic circulation, OVs can undergo filtration and accumulate in specific tissues, primarily the liver. In addition, OVs present in the bloodstream can be recognized by the immune system and eliminate them. Furthermore, OVs must overcome the tumor vasculature and interstitial fluid pressure to enter the extracellular space. Ultimately, the tumor microenvironment poses various obstacles that restrict viral penetration and propagation [26–28]. Several strategies, including the use of cell carriers, are being developed to overcome these challenges [26]. Using cell carriers as a vehicle for systemic transport of OVs to both primary tumors and metastases is an appealing strategy. This is because cell carriers can provide protection to OVs from complement or neutralizing antibodies, which are the principal means of viral clearance in the bloodstream [29, 30]. Furthermore, administering cell-loaded OVs systemically can circumvent filtration by organs and cross the endothelial barrier via migration towards various tissues and organs [31]. Finally, certain types of cells have attracted attention due to their unique ability to specifically migrate toward tumors. These cell types include immune cells, progenitor cells, cancer cells, neural stem cells, extracellular vesicles (EVs), and MSCs. Out of these, MSCs are especially intriguing because of their inherent characteristics [32, 33].

OVs can potentially be delivered to cancer cells through MSCs. There are different strategies for using MSCs to deliver OVs, but one approach involves engineering the MSCs to express and secrete the OV. The modified MSCs can then be injected into the patient’s bloodstream, where they will migrate toward the tumor site. Once at the tumor site, the MSCs can release the OV directly into the tumor microenvironment, allowing it to infect and kill the cancer cells. This approach has several potential advantages, including increased targeting of the OV to the tumor, reduced toxicity to healthy tissues, and the ability of MSCs to home in on sites of inflammation. However, there are also challenges associated with this approach, such as the need to optimize the delivery of the MSCs and ensure that they survive long enough to release the OV within the tumor microenvironment. Additionally, there is a risk that the MSCs themselves could contribute to tumor growth or metastasis if they are not fully characterized and controlled. The loading capacity of MSCs with OVs can vary depending on several factors such as the type of virus being used, the MOI (multiplicity of infection) used during loading, and the size of the MSCs. Generally, studies have reported that MSCs can effectively deliver and release OVs, with loading capacities ranging from 10 to 1000 viral particles per cell.

#### III. Trojanhorse strategy

**Fig. 1:**
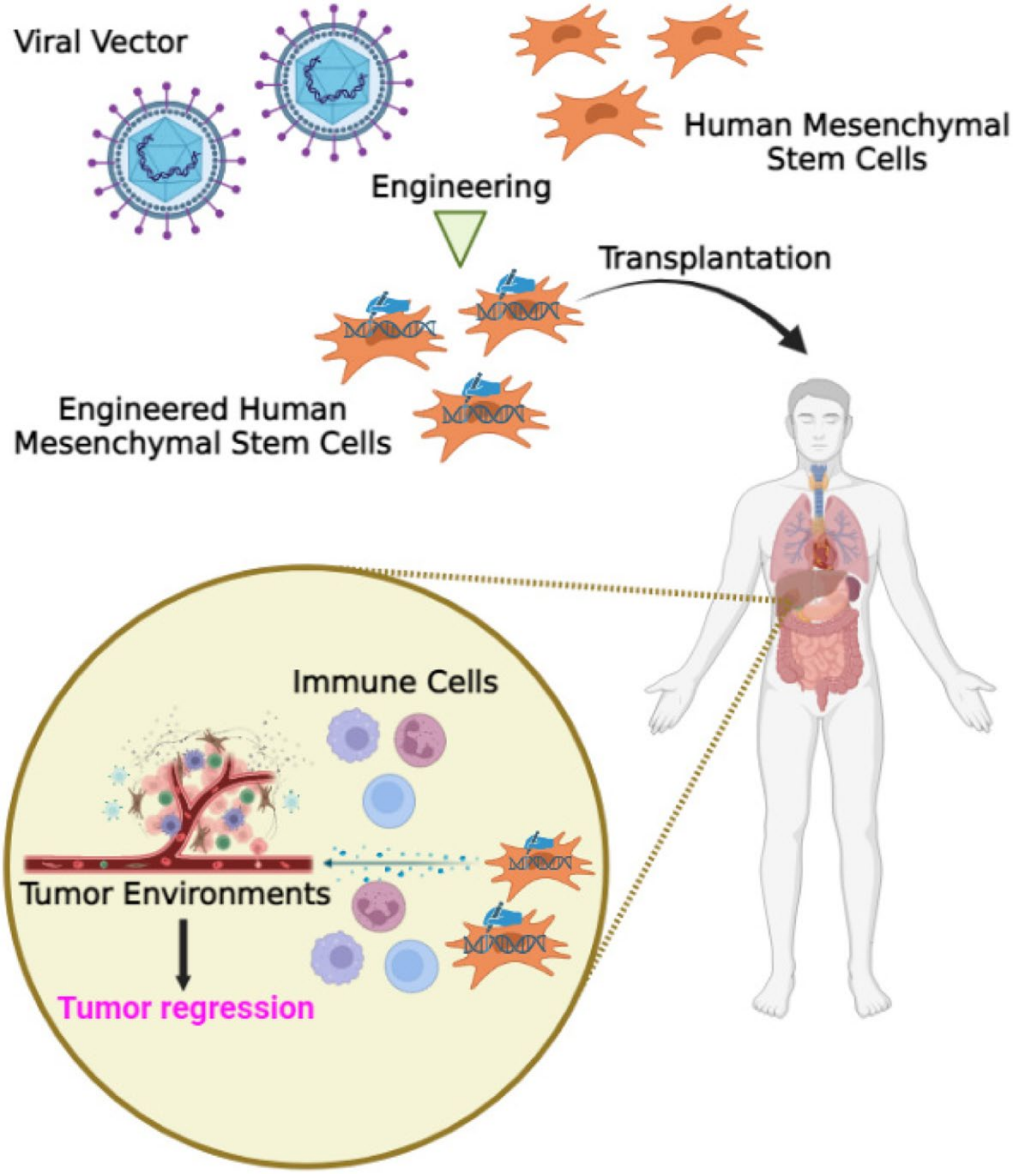
MSC-based delivery for tumor immunotherapy

Recent preclinical studies have shown that MSCs are effective carriers for OVs, providing a promising approach for improving the efficiency of oncolytic virotherapy [34]. MSCs possess several characteristics that make them an ideal alternative for OV chaperoning in cancer treatment, including their inherent anticancer properties, tumor-homing ability, ability to preserve OVs from neutralizing antibodies, and capacity to distribute OVs to tumor sites via a Trojan horse strategy [35, 36]. Numerous studies have demonstrated that MSCs can migrate to sites of injury, ischemia, and tumors through chemotaxis, although the precise mechanisms of their migration remain unclear [37]. Various cytokine/receptor pairs, including SDF-1/CXCR4, PDGF/PDGFR, HGF/c-Met, VEGF/VEGFR, SCF/c-Kit, HMGB1/RAGE, and MCP-1/CCR2, regulate MSC migration [38, 39]. In addition, soluble molecules released by immune cells and cancer cells within the tumor microenvironment (TME) can directly influence MSC chemotaxis [40]. In addition, the efficiency of MSC migration to these sites can be influenced by factors such as the vascularization, oxidative state, and inflammatory condition of the tumor. As an example, interleukin-6 (IL-6) can enhance MSC attraction to cancerous areas, whereas MSC migration in glioma is dependent on IL-8. TME plays a crucial role in cancer progression and treatment response. Recent studies have also demonstrated that MSCs can regulate TME by modulating immune cell function, angiogenesis, extracellular matrix remodeling, and inflammation. Specifically, MSCs can secrete factors such as cytokines, chemokines, growth factors, and extracellular vesicles that can influence the behavior of neighboring cells within TME [41, 42]. Furthermore, tumors may attract MSCs from other tissues such as adipose tissue (AD-MSCs) and bone marrow (BM-MSCs) and encourage their integration into the tumor microenvironment through inflammatory signals [41, 42]. Factors present in the local environment, such as hypoxia, Toll-like receptor (TLR), and cytokines ligands, can activate the recruited MSCs to proliferate and secrete growth factors that augment tissue regeneration [43, 44]. MSCs have been shown to be attracted to glioma, breast cancer, and hepatic carcinoma (Fig. 1) [45–47].

Utilizing MSCs as carriers for cellular delivery for drugs offers several advantages, including site-specificity and the potential for tailored, constant drug release, which can circumvent biological drug half-life limitations [48–50]. This approach can also address the challenge of maintaining effective drug concentrations near tumors for extended periods, Especially when it comes to brain tumors, substances may not be able to pass through the blood-brain barrier [50]. Using MSCs as OV carriers is a new strategy to overcome these challenges, which allows for a more accurate and long-lasting therapeutic response compared to traditional delivery methods. Furthermore, MSCs can also act as biological factories for viral genome replication, resulting in an increased virus titer [51]. As a result, even a small initial amount of OVs loaded into MSCs can deliver a substantial viral dose to the tumor microenvironment. Nonetheless, the mechanisms by which OV replication occurs within MSCs are yet to be understood. When MSCs transport OVs, they leverage their inherent attraction for reaching tumor sites, thereby enhancing OV homing and facilitating oncolysis [52].

Utilizing MSCs as carriers for OVs involves a complex interplay of molecular interactions that facilitate their migration to tumor sites. Several key mechanisms have been identified that contribute to the transport of OVs by MSCs, enhancing their tumor-homing ability and facilitating effective oncolysis. MSC migration is primarily driven by chemotaxis, a process wherein cells respond to chemical gradients. Various cytokine/receptor pairs play a crucial role in regulating MSC migration towards tumor sites. Examples include the stromal cell-derived factor-1 (SDF-1) and its receptor CXCR4, platelet-derived growth factor (PDGF) and its receptor PDGFR, hepatocyte growth factor (HGF) and c-Met receptor, vascular endothelial growth factor (VEGF) and VEGF receptor (VEGFR), stem cell factor (SCF) and c-Kit receptor, high-mobility group box 1 (HMGB1) and receptor for advanced glycation end products (RAGE), and monocyte chemoattractant protein-1 (MCP-1) and its receptor CCR2. These interactions regulate the migration of MSCs towards the tumor microenvironment (TME), guided by the chemotactic signals released by immune cells and cancer cells within the TME. The local environment within the tumor, characterized by factors like hypoxia, Toll-like receptor (TLR) ligands, and cytokines, plays a crucial role in activating and recruiting MSCs. Inflammatory signals can attract MSCs from other tissues, such as adipose tissue (AD-MSCs) and bone marrow (BM-MSCs), to integrate into the TME. Factors like interleukin-6 (IL-6) and IL-8 released by the tumor can enhance MSC attraction to cancerous areas and glioma, respectively. Additionally, the vascularization, oxidative state, and inflammatory condition of the tumor influence the efficiency of MSC migration to tumor sites. Cell adhesion molecules (CAMs) play a vital role in mediating the interaction between MSCs and the components of the tumor microenvironment. CAMs such as integrins, selectins, and cadherins are involved in the adhesion of MSCs to endothelial cells, extracellular matrix proteins, and tumor cells. These interactions facilitate the migration and extravasation of MSCs towards tumor sites. EVs released by MSCs contribute to intercellular communication and play a role in their migration to tumor sites. These small membrane-bound vesicles carry bioactive molecules, including proteins, nucleic acids, and lipids, which can modulate the tumor microenvironment and promote tumor-homing of MSCs. MSCs possess inherent tumor-homing ability, although the precise mechanisms behind this phenomenon are not yet fully understood. The migration of MSCs towards tumors may involve a combination of chemotactic signals, receptor-ligand interactions, and the unique characteristics of the tumor microenvironment.

### Suppressive effects of mscs on the immune system

Several studies have shown that MSCs possess immunosuppressive and anti-inflammatory properties [52–54]. Research has indicated that MSCs can mitigate renal damage in diabetic nephropathy by inhibiting CD103 + DCs and CD68 + CD11c + macrophages within the kidneys [55]. Furthermore, MSCs have the ability to hinder the maturation of CD14 + monocytes and CD34 + progenitor cells into mature DCs, thus constraining both the differentiation and functionality of DCs, and promote the development of regulatory immune subtypes, such as CD4 + CD25 + FOXP3 + T lymphocytes (Tregs), IL-10-producing B lymphocytes, CD8 + CD28-T lymphocytes, and IL-10-producing DCs [56, 57].

In addition, MSCs secrete a diverse range of soluble cytokines, such as IL-6, IL-10, TGF-β1, heme oxygenase-1 (HO-1), inducible nitric oxide synthase (iNOS), and indoleamine-2-dioxygenase-3 (IDO), which are crucial in impeding the immune response [58]. MSCs’ cytokine secretion can impede B lymphocyte maturation, diminish their ability to produce immunoglobulin, hinder helper T cells from releasing cytokines, lower the cytotoxicity of effector T lymphocytes, and suppress NK cell proliferation, cytokine production, and cytotoxicity [59]. The immune modulatory characteristics of MSCs play a vital role in suppressing inflammation in the vicinity during virotherapy. This, in turn, enables the OV to proliferate and eliminate cancer cells unobstructed by the immune system [60]. The immunosuppressive effects of MSCs in oncolytic virotherapy OV are crucial. MSCs enhance viral replication and oncolysis by suppressing immune cells, enabling efficient virus spread within the tumor microenvironment. They also produce factors that induce cancer cell apoptosis, further promoting OV efficacy. MSCs mitigate immune responses against OV by inhibiting immune cell activation, reducing inflammation, and modulating the functions of dendritic cells and macrophages. However, context-dependent factors must be considered, and a balance between enhancing OV effectiveness and preserving an effective anti-cancer immune response is essential [53, 54].

### MSC properties inhibiting tumor growth

Numerous studies have confirmed that MSCs can hinder the growth of tumors [61]. By functioning as a pro-inflammatory agent, the combination of MSCs and tumor cells stimulates the influx of monocytes, granulocytes, and T lymphocytes, thereby promoting intercellular communication between these immune cells and adjacent tissues [62]. This process leads to the generation of various chemokines by the immune cells and the inflamed tissues, which can attract activated lymphocytes through the corresponding receptors, further stimulating anti-cancer immune responses [62]. Multiple investigations have reported that oncolytic virus-loaded MSCs specifically target tumor xenografts, resulting in reduced tumor sizes and improved survival rates in treated animals. However, the effect of MSCs on tumor growth appears to vary depending on several factors, such as tumor type, MSC preparation, as well as the duration and amount of MSC administration [63]. Researchers conducted a study comparing the effectiveness of modified MSCs called MSCs-E1s to unmodified MSCs in delivering adenoviral vectors for tumor treatment. MSCs-E1s, which contained essential genes for adenoviral replication, demonstrated superior antitumor activity in laboratory experiments compared to unmodified MSCs [64]. The presence of E1s in MSCs-E1s facilitated a higher production of progeny vectors, resulting in a greater number of infected and killed tumor cells. Further analysis using mice models with prostate tumors revealed that both genetically modified MSCs loaded with conditionally replicating adenoviruses (CRAds) and MSCs loaded with Adbic (a therapeutic gene) successfully packaged, replicated, and delivered the adenoviral vectors to the tumor site. MSCs-E1s displayed a preference for integrating into the tumors, effectively spreading within the tumor environment and suppressing the growth of prostate tumors in the mice models [63]. These findings suggest that utilizing adenoviral vector-loaded MSCs-E1s holds promise as a targeted and efficient approach for treating prostate tumors. Although MSCs have been found to boost the growth of certain tumor cell lines in vivo, they can also restrict tumor progression by interfering with the cell cycle, suppressing the PI3K/AKT pathway, and expressing suppressor genes [65, 66]. For example, in a murine model of melanoma, bone marrow-derived MSCs demonstrated cytotoxic effects on tumors by generating reactive oxygen species when interacting with capillary endothelial cells, resulting in delayed tumor growth and apoptosis of endothelial cells. However, these cytotoxic effects of MSCs were only noticeable in significant quantities [67]. In a different investigation, using a mouse model of breast cancer metastasis, researchers discovered that umbilical cord-derived MSCs (UC-MSC) and AD-MSCs could impede lung metastasis and decelerate tumor progression by inciting apoptosis through PARP and caspase-3 cleavage [68].

In a murine model of Kaposi sarcoma, human MSCs (hMSCs) were injected intravenously and successfully targeted cancer sites, leading to a substantial reduction in tumorigenesis by impeding Akt signaling and E-cadherin-mediated cell contact. Additionally, in vitro and melanoma murine models have demonstrated that MSCs display anti-angiogenic traits. However, the effects of AD-MSCs are seemingly contradictory in breast and prostate cancer as they exhibit both pro- and anti-cancer abilities [69, 70]. In the presence of tumor cells, MSCs function as a pro-inflammatory factor by amplifying the infiltration of monocytes, granulocytes, and T lymphocytes into the neighboring tissues. This heightened infiltration augments communication between the immune cells and surrounding tissues, resulting in the generation of various chemokines that lure activated lymphocytes possessing corresponding receptors, ultimately promoting anti-cancer immunity. However, the functional impact of MSCs on tumor growth may fluctuate based on several factors, underscoring the significance of further research [71–73]. In vitro, studies with the murine C3H10T1/2 (C3) MSC line and primary MSCs demonstrated their immunosuppressive properties in a mixed lymphocyte reaction. This immunosuppressive effect was mediated by soluble factors secreted by activated MSCs in the presence of splenocytes. CD8 + regulatory cells were identified as being responsible for inhibiting allogeneic lymphocyte proliferation, thereby contributing to immunosuppression. Furthermore, it was observed that implantation of C3 MSCs expressing the human bone morphogenetic protein 2 (hBMP-2) differentiation factor into various allogeneic immunocompetent mice was not rejected and was still capable of differentiating into bone. The study also investigated the impact of MSCs on tumor growth using a murine melanoma tumor model. It was found that subcutaneous injection of B16 melanoma cells led to tumor growth specifically in allogeneic recipients when MSCs were co-injected [74].

###  Mechanisms of oncolytic viruses for cancer treatment

Tumor cells possess dissimilar genetic and physiological characteristics compared to normal cells, such as abnormal antiviral capability and signaling pathways, tumor-specific promoters or mRNAs, and distinct expression of genes related to apoptosis or surface receptor proteins [75]. These features enable OVs to specifically target tumor cells without reproducing in normal cells, resulting in their safe usage for clinical purposes. The IFN pathway, an antiviral signaling pathway in healthy cells, can eliminate viruses. However, malfunctions and deletions in critical protein-coding genes of these signaling pathways in tumor cells alter the pathways, leading to the promotion of viral replication, infection, and dissemination [76]. As an example, the matrix (M) protein of Vesicular stomatitis virus (VSV) performs a crucial function in virus assembly and repression of host gene expression. Variants of the M protein can specifically propagate in tumor cells having impaired IFN immune responses but are unable to do so in healthy tissue cells due to the activation of the IFN signaling pathway [77, 78]. Likewise, the activation of ELK, a downstream protein of RAS, can regulate the expression of ICP4, a critical protein essential for HSV replication. This enables a mutant HSV to specifically reproduce in tumor cells where the RAS signaling pathway is activated [79, 80]. Reovirus, a wild-type virus, can naturally target tumor cells with hyperactive RAS without requiring modification. In oncolytic virotherapy, some promoters can regulate the expression of crucial viral replication genes, thus restricting virus propagation in tumor tissues [81]. Employing promoters like E2F-1, hTERT, and HIF-1, which exhibit high expression levels in tumor tissues but low levels in normal tissues, can intensify the virus’s specificity. By positioning crucial viral replication genes like E1A and E1B under these promoters, the genes’ expression is triggered in tumor tissues [82].

In addition, tissue-specific miRNA sequences can be incorporated into the genome of the oncolytic virus to regulate the expression of critical genes, thereby inhibiting virus reproduction in normal tissue cells. For instance, miR199 is observed to be downregulated in hepatocellular carcinoma (HCC) compared to normal liver tissues. As a result, Adenovirus-199T (Ad-199T), a genetically modified adenovirus, has copies of DNA segments complementary to miR199 introduced into the crucial adenovirus replication gene E1A. This insertion allows Ad-199T to propagate in cells devoid of miR-199, thereby inducing virus replication and cytolytic effects that are exclusively selective for HCC cells [83].

 To ensure viral selectivity, targeting essential genes for viral replication in normal cells that are irrelevant to viral reproduction in tumors is a feasible approach. The E1B-55 K protein, which prevents premature apoptosis, is expressed in natural adenoviruses. In healthy cells, the p53 gene is a critical apoptosis-inducing gene, whereas inactivation of the p53 gene is integral to tumor cells. Viral replication hinges on host cell apoptosis inhibition, which is connected to p53 function. Hence, an oncolytic adenovirus with an E1B gene deletion cannot reproduce in normal cells due to the loss of apoptosis inhibition, whereas its replication in p53-deficient tumor cells remains unaffected. Another method of rendering a virus selective to tumors involves genetically modifying the viral capsid protein to allow binding exclusively to tumor cells [84]. The MV-h-uPA construct comprises an added fragment that binds to uPAR, a receptor with high expression levels on the surface of various tumor cells, closely associated with tumor invasiveness and angiogenesis (Table 1). This receptor-ligand pathway allows for selective infection of tumor cells exhibiting elevated uPAR expression. Furthermore, incorporating lysine residues into ciliated proteins permits virus binding to tumor cells with widespread heparin sulfate receptors. For example, HER-2 is overexpressed in 15–20% of mammary and ovarian carcinomas. By substituting the Ig-folded core in the receptor-binding virion glycoprotein D with an anti-HER-2 single-chain antibody, HSVs can be modified to target HER2. Combining engineered viral glycoproteins, gD and gB, enables HSVs to trigger highly efficient infection in glioblastoma cells that exhibit high EGFR expression (Table 2) [85–89]. Two major challenges include immune system-mediated viral clearance and barriers in the tumor microenvironment that restrict viral penetration and propagation. To overcome these challenges, potential technologies to explore include immunomodulatory strategies, tumor microenvironment modulation, combination therapies, genetically engineered OVs, and virotherapy delivery systems [90, 91].

**Table 1:** MSC delivery of OVs in different cancer

Cancer	OV	MSC type	Delivery method	References
Hepatocellular carcinoma (HCC)	Adenovirus	Bone marrow-derived MSCs	Intratumoral injection	[83]
Ovarian cancer	Newcastle disease virus	Adipose-derived MSCs	Intraperitoneal injection	[26]
Breast cancer	Vesicular stomatitis virus	Umbilical cord blood-derived MSCs	Intratumoral injection	[68]
Glioblastoma	Measles virus	Bone marrow-derived MSCs	Intracranial injection	[49]
Prostate cancer	Herpes simplex virus-1	Adipose-derived MSCs	Intratumoral injection	[69]

**Table 2:** Oncolytic viruses and their mechanisms

Virus	Viral Protein	Mechanism	Reference
Vesicular stomatitis virus (VSV)	Matrix (M) protein	Replication in tumor cells with defective IFN immune responses	[78]
	ICP4 Protein	Selective replication in tumor cells with activated RAS signaling pathway	[80]
Mutant Herpes Simplex Virus (HSV)	Engineered glycoproteins gD and gB	Highly efficient infection in glioblastoma cells with high EGFR expression	
Reovirus	NA	Natural targeting of tumor cells with overactive RAS	[81]
Recombinant Adenovirus (Ad-199T)	DNA segments complementary to miR199 inserted within E1A gene	Replication in cells lacking miR-199 in hepatocellular carcinoma (HCC)	[83]
Oncolytic Adenovirus with E1B gene deletion	E1B-55 K protein	Inability to replicate in normal cells due to dysfunction of apoptosis inhibition	[84]
Modified Measles Virus (MV-h-uPA)	Fragment added that binds to uPAR receptor	Specific infection of tumor cells with high uPAR expression	[85]
Modified Herpes Simplex Virus (HSV)	Anti-HER-2 single-chain antibody, gD and gB glycoproteins	Targets HER2 and mediates highly efficient infection in glioblastoma cells with high EGFR expression	[86]

### Future directions and potential areas of research

The investigation of OVs delivered via MSCs for cancer treatment shows great promise and calls for further research. Future directions include optimizing delivery platforms, evaluating safety profiles, exploring combination therapies with other modalities, understanding immunomodulation and immune responses, conducting preclinical and clinical studies, and assessing scalability and cost-effectiveness. These efforts aim to enhance targeting efficiency, mitigate risks, improve therapeutic outcomes, elucidate mechanisms, validate efficacy and safety in humans, and pave the way for personalized cancer treatments. Continued exploration of this strategy holds significant potential for revolutionizing cancer therapeutics.

## Conclusion

In conclusion, the combination of MSCs with OVs presents a promising and targeted approach to cancer treatment. The utilization of MSCs’ migratory and tumor-targeting properties, along with the tumor cell-destroying abilities of OVs, makes this strategy suitable for both localized and metastatic malignancies. Furthermore, the ability of OVs to induce immunogenic cell death and trigger anti-tumor immune responses enhances the therapeutic potential. Importantly, the combination of MSCs and OVs also offers the opportunity to modulate the tumor microenvironment, augmenting the effectiveness of immunotherapies. However, certain challenges remain to be addressed. Optimization of delivery procedures, improvement of safety profiles, and enhancing OV efficiency are crucial aspects that require attention. Further studies are necessary to determine the optimal timing, dosage, and frequency of administering MSC-releasing OVs in different cancer types and stages. The prospects of MSC-releasing OVs appear promising, but their efficacy and safety must be validated through additional research and clinical trials. This comprehensive evaluation underscores the potential benefits of MSC-releasing OVs while acknowledging the importance of ongoing investigation in this field.

## Data Availability

Not applicable.

## References

[1] Schillaci O, Scimeca M, Toschi N, Bonfiglio R, Urbano N, Bonanno E (2019). Combining diagnostic imaging and pathology for improving diagnosis and prognosis of cancer. Contrast Media Mol Imaging..

[2] -Huang S, Yang J, Fong S, Zhao Q (2020). Artificial intelligence in cancer diagnosis and prognosis: Opportunities and challenges. Cancer letters. Feb.

[3] Navya PN, Kaphle A, Srinivas SP, Bhargava SK, Rotello VM, Daima HK (2019). Current trends and challenges in cancer management and therapy using designer nanomaterials. Nano Convergence..

[4] Trédan O, Galmarini CM, Patel K, Tannock IF (2007). Drug resistance and the solid tumor microenvironment. J Nat Cancer Institute..

[5] Mokhtari RB, Homayouni TS, Baluch N, Morgatskaya E, Kumar S, Das B, Yeger H (2017). Combination therapy in combating cancer. Oncotarget.

[6] Soltani S, Tabibzadeh A, Yousefi P, Zandi M, Zakeri A, Akhavan Rezayat S, Ramezani A, Esghaei M, Farahani A (2021). HPV infections in retinoblastoma: a systematic review. J Clin Lab Anal..

[7] Lind MJ (2020). Principles of systemic anticancer therapy. Medicine..

[8] Qian CN, Mei Y, Zhang J (2017). Cancer metastasis: issues and challenges. Chin J Cancer..

[9] Bădilă AE, Rădulescu DM, Niculescu AG, Grumezescu AM, Rădulescu M, Rădulescu AR (2021). Recent advances in the treatment of bone metastases and primary bone tumors: An up-to-date review. Cancers..

[10] Sorkhabi AD, Sarkesh A, Saeedi H, Marofi F, Ghaebi M, Silvestris N, Baradaran B, Brunetti O (2022). The basis and advances in clinical application of cytomegalovirus-specific cytotoxic T cell immunotherapy for glioblastoma multiforme. Front Oncol..

[11] Cobbs CS (2013). Cytomegalovirus and brain tumor: epidemiology, biology and therapeutic aspects. Curr Opin Oncol.

[12] Jhawar SR, Thandoni A, Bommareddy PK, Hassan S, Kohlhapp FJ, Goyal S, Schenkel JM, Silk AW, Zloza A (2017). Oncolytic viruses—natural and genetically engineered cancer immunotherapies. Front Oncol..

[13] Subramaniam DS, Liu SV, Giaccone G (2016). Novel approaches to Cancer Immunotherapy. Discovery Med.

[14] Engeland CE, Bell JC, Engeland C (2020). Introduction to Oncolytic Virotherapy. Oncolytic Viruses. Methods in Molecular Biology.

[15] Romero D (2018). Oncolytic viruses prime antitumour immunity. Nat Rev Clin Oncol..

[16] Ylösmäki E, Cerullo V (2020). Design and application of oncolytic viruses for cancer immunotherapy. Current Opinion Biotechnol..

[17] de Gruijl TD, Janssen AB, van Beusechem VW (2015). Arming oncolytic viruses to leverage antitumor immunity. Expert Opinion Biol Ther..

[18] Liu Y, Kim YJ, Siriwon N, Rohrs JA, Yu Z, Wanga P (2018). Combination drug delivery via multilamellar vesicles enables targeting of tumor cells and tumor vasculature. Biotechnol Bioeng..

[19] Hendijani F, Javanmard SH (2015). Dual protective and cytotoxic benefits of mesenchymal stem cell therapy in combination with chemotherapy/radiotherapy for cancer patients. Crit Rev Eukaryot Gene Expr..

[20] Zou W, Zheng H, He TC, Chang J, Fu YX, Fan W (2012). LIGHT delivery to tumors by mesenchymal stem cells mobilizes an effective Antitumor Immune ResponseReversing suppressive Tumor Microenvironment by MSC-L. Cancer Res..

[21] Seyed-Khorrami SM, Soleimanjahi H, Soudi S, Habibian A (2021). MSCs loaded with oncolytic reovirus: migration and in vivo virus delivery potential for evaluating anti-cancer effect in tumor-bearing C57BL/6 mice. Cancer Cell Int..

[22] Aithal AP, Bairy LK, Seetharam RN (2017). Safety assessment of human bone marrow-derived mesenchymal stromal cells transplantation in Wistar rats. J Clin Diagn Res..

[23] Park D, Spencer JA, Koh BI, Kobayashi T, Fujisaki J, Clemens TL, Lin CP, Kronenberg HM, Scadden DT (2012). Endogenous bone marrow MSCs are dynamic, fate-restricted participants in bone maintenance and regeneration. Cell Stem Cell..

[24] Niemeyer P, Vohrer J, Schmal H, Kasten P, Fellenberg J, Suedkamp NP, Mehlhorn AT (2008). Survival of human mesenchymal stromal cells from bone marrow and adipose tissue after xenogenic transplantation in immunocompetent mice. Cytotherapy..

[25] Zhu J, Inomata T, Fujimoto K, Uchida K, Fujio K, Nagino K, Miura M, Negishi N, Okumura Y, Akasaki Y, Hirosawa K (2021). Ex vivo–induced bone marrow-derived myeloid suppressor cells prevent corneal allograft rejection in mice. Investig Ophthalmol Visual Sci..

[26] Mohamadi A, Hashemzadeh MS (2020). The Important Role of Oncolytic Viruses in Common Cancer Treatments. Current Cancer Ther Rev..

[27] An Y, Yang Q (2021). Tumor-associated macrophage-targeted therapeutics in ovarian cancer. Int J Cancer.

[28] Gujar S, Bell J, Diallo JS (2019). SnapShot: cancer immunotherapy with oncolytic viruses. Cell..

[29] Ilett E, Kottke T, Donnelly O, Thompson J, Willmon C, Diaz R, Zaidi S, Coffey M, Selby P, Harrington K, Pandha H (2014). Cytokine conditioning enhances systemic delivery and therapy of an oncolytic virus. Mol Ther..

[30] Zhong P, Agosto LM, Munro JB, Mothes W (2013). Cell-to-cell transmission of viruses. Current OpinionVirology..

[31] Nishida-Aoki N, Tominaga N, Kosaka N, Ochiya T (2020). Altered biodistribution of deglycosylated extracellular vesicles through enhanced cellular uptake. J Extracellular Vesicles..

[32] Li HJ, Reinhardt F, Herschman HR, Weinberg RA (2012). Cancer-Stimulated Mesenchymal Stem Cells Create a Carcinoma Stem Cell Niche via Prostaglandin E2 SignalingPGE2 and the MSC-Derived Cancer Stem Cell Niche. Cancer Discovery..

[33] Wang J, Chen Z, Sun M, Xu H, Gao Y, Liu J, Li M (2020). Characterization and therapeutic applications of mesenchymal stem cells for regenerative medicine. Tissue Cell..

[34] Reale A, Calistri A, Altomonte J (2021). Giving oncolytic viruses a free ride: carrier cells for oncolytic virotherapy. Pharmaceutics..

[35] Ferguson SD, Ahmed AU, Thaçi B, Mercer RW, Lesniak MS (2010). Crossing the boundaries: stem cells and gene therapy. Discov Med..

[36] Mader EK, Maeyama Y, Lin Y, Butler GW, Russell HM, Galanis E, Russell SJ, Dietz AB, Peng KW (2009). Mesenchymal stem cell carriers protect oncolytic measles viruses from antibody neutralization in an Orthotopic Ovarian Cancer Therapy ModelVirotherapy in Immune mice using MSC Cell Carriers. Clin Cancer Res.

[37] Salmasi Z, Hashemi M, Mahdipour E, Nourani H, Abnous K, Ramezani M (2020). Mesenchymal stem cells engineered by modified polyethylenimine polymer for targeted cancer gene therapy, in vitro and in vivo. Biotechnol Prog..

[38] Son BR, Marquez-Curtis LA, Kucia M, Wysoczynski M, Turner AR, Ratajczak J, Ratajczak MZ, Janowska‐Wieczorek A (2006). Migration of bone marrow and cord blood mesenchymal stem cells in vitro is regulated by stromal‐derived factor‐1‐CXCR4 and hepatocyte growth factor‐c‐met axes and involves matrix metalloproteinases. Stem Cells.

[39] Momin N, Vela E, Zaidi GA, Quiñones-Hinojosa HA (2010). The oncogenic potential of mesenchymal stem cells in the treatment of cancer: directions for future research. Curr Immunol Rev..

[40] Riemann A, Ihling A, Reime S, Gekle M, Thews O. Impact of the tumor microenvironment on the expression of inflammatory mediators in cancer cells. InOxygen Transport to tissue XXXVIII 2016 (pp. 105–11). Springer International Publishing. 10.1007/978-3-319-38810-6_14 27526131

[41] -Shan Y, He X, Song W, Han D, Niu J, Wang J (2015). Role of IL-6 in the invasiveness and prognosis of glioma. Int J Clin Exp Med.

[42] Ringe J, Strassburg S, Neumann K, Endres M, Notter M, Burmester GR, Kaps C, Sittinger M (2007). Towards in situ tissue repair: human mesenchymal stem cells express chemokine receptors CXCR1, CXCR2 and CCR2, and migrate upon stimulation with CXCL8 but not CCL2. J Cellul Biochem..

[43] Marquez-Curtis LA, Janowska-Wieczorek A (2013). Enhancing the migration ability of mesenchymal stromal cells by targeting the SDF-1/CXCR4 axis. Biomed Res Int..

[44] Rustad KC, Gurtner GC (2012). Mesenchymal stem cells home to sites of injury and inflammation. Adv Wound Care..

[45] Hai C, Jin YM, Jin WB, Han ZZ, Cui MN, Piao XZ, Shen XH, Zhang SN, Sun HH (2012). Application of mesenchymal stem cells as a vehicle to deliver replication-competent adenovirus for treating malignant glioma. Chin J Cancer..

[46] Ma F, Chen D, Chen F, Chi Y, Han Z, Feng X, Li X, Han Z (2015). Human umbilical cord mesenchymal stem cells promote breast cancer metastasis by interleukin-8-and interleukin-6-dependent induction of CD44+/CD24-cells. Cell Transplant..

[47] Xie C, Yang Z, Suo Y, Chen Q, Wei D, Weng X, Gu Z, Wei X (2017). Systemically infused mesenchymal stem cells show different homing profiles in healthy and tumor mouse models. Stem Cells Transl Med..

[48] McCrea Z, Arnanthigo Y, Cryan SA, O’Dea S (2018). A novel methodology for bio-electrospraying mesenchymal stem cells that maintains differentiation, immunomodulatory and pro-reparative functions. J Med Biol Eng..

[49] Ali S, Xia Q, Muhammad T, Liu L, Meng X, Bars-Cortina D, Khan AA, Huang Y, Dong L (2021). Glioblastoma therapy: rationale for a mesenchymal stem cell-based vehicle to carry recombinant viruses. Stem Cell Rev Rep.

[50] Tahir M, Ahmad N, Lei D, Ali S (2022). Emerging role of oncolytic viruses and stem cells in gene therapy: Should they be integrated?. Drug Discov Today..

[51] Khalili M (2022). Evaluation of the survival rate and clinical outcome of nanodrug administration for the treatment of lung cancer: a systematic review and meta-analysis. Int J Sci Res Dent Med Sci.

[52] Hakkarainen T, Särkioja M, Lehenkari P, Miettinen S, Ylikomi T, Suuronen R, Desmond RA, Kanerva A, Hemminki A (2007). Human mesenchymal stem cells lack tumor tropism but enhance the antitumor activity of oncolytic adenoviruses in orthotopic lung and breast tumors. Hum Gene Ther..

[53] Bouffi C, Bony C, Courties G, Jorgensen C, Noel D (2010). IL-6-dependent PGE2 secretion by mesenchymal stem cells inhibits local inflammation in experimental arthritis. PloS One..

[54] Chaudhary D, Trivedi RN, Kathuria A, Goswami TK, Khandia R, Munjal A (2018). In vitro and in vivo immunomodulating properties of mesenchymal stem cells. Recent Patents Inflamm Allerg Drug Discov..

[55] Ullah I, Subbarao RB, Rho GJ (2015). Human mesenchymal stem cells-current trends and future prospective. Biosci Rep..

[56] Lv SS, Liu G, Wang JP, Wang WW, Cheng J, Sun AL, Liu HY, Nie HB, Su MR, Guan GJ (2013). Mesenchymal stem cells transplantation ameliorates glomerular injury in streptozotocin-induced diabetic nephropathy in rats via inhibiting macrophage infiltration. Int Immunopharmacol..

[57] Liu WH, Liu JJ, Wu J, Zhang LL, Liu F, Yin L, Zhang MM, Yu B (2013). Novel mechanism of inhibition of dendritic cells maturation by mesenchymal stem cells via interleukin-10 and the JAK1/STAT3 signaling pathway. PLoS ONE..

[58] Negi N, Griffin MD (2020). Effects of mesenchymal stromal cells on regulatory T cells: current understanding and clinical relevance. Stem Cells..

[59] Liang C, Jiang E, Yao J, Wang M, Chen S, Zhou Z, Zhai W, Ma Q, Feng S, Han M (2018). Interferon-γ mediates the immunosuppression of bone marrow mesenchymal stem cells on T-lymphocytes in vitro. Hematology..

[60] Cargnoni A, Romele P, Bonassi Signoroni P, Farigu S, Magatti M, Vertua E, Toschi I, Cesari V, Silini AR, Stefani FR, Parolini O (2020). Amniotic MSCs reduce pulmonary fibrosis by hampering lung B-cell recruitment, retention, and maturation. Stem Cells Transl Med..

[61] Mardi A, Shirokova AV, Mohammed RN, Keshavarz A, Zekiy AO, Thangavelu L, Mohamad TA, Marofi F, Shomali N, Zamani A, Akbari M (2022). Biological causes of immunogenic cancer cell death (ICD) and anti-tumor therapy; combination of Oncolytic virus-based immunotherapy and CAR T-cell therapy for ICD induction. Cancer Cell Int..

[62] Zhao R, Chen X, Song H, Bie Q, Zhang B (2020). Dual role of MSC-derived exosomes in tumor development. Stem Cells Int..

[63] Hussain Z, Rahim MA, Jan N, Shah H, Rawas-Qalaji M, Khan S, Sohail M, Thu HE, Ramli NA, Sarfraz RM, Abourehab MA (2021). Cell membrane cloaked nanomedicines for bio-imaging and immunotherapy of cancer: improved pharmacokinetics, cell internalization and anticancer efficacy. J Controlled Release..

[64] Babaei A, Soleimanjahi H, Soleimani M, Arefian E (2021). Mesenchymal stem cells loaded with oncolytic reovirus enhances antitumor activity in mice models of colorectal cancer. Biochem Pharmacol..

[65] Muhammad T, Sakhawat A, Khan AA, Ma L, Gjerset RA, Huang Y (2019). Mesenchymal stem cell-mediated delivery of therapeutic adenoviral vectors to prostate cancer. Stem Cell Res Ther..

[66] -Wang R, Lu X, Yu R (2020). lncRNA MALAT1 promotes EMT process and cisplatin resistance of oral squamous cell carcinoma via PI3K/AKT/m-TOR signal pathway. OncoTargets Ther.

[67] Zandi M, Shokri S, Mahmoudvand S, Hosseinzadeh Adli A, Mohammadi R, Haddadi A (2022). Interplay between cellular metabolism and DNA viruses. J Med Virol..

[68] Yang C, Zhang Y, Lin S, Liu Y, Li W (2021). Suppressing the KIF20A/NUAK1/Nrf2/GPX4 signaling pathway induces ferroptosis and enhances the sensitivity of colorectal cancer to oxaliplatin. Aging (Albany NY)..

[69] -Sun B (2009). Therapeutic potential of mesenchymal stromal cells in a mouse breast cancer metastasis model. Cytotherapy.

[70] Otsu K (2009). Concentration-dependent inhibition of angiogenesis by mesenchymal stem cells. Blood J Am Soc Hematol.

[71] Khakoo AY (2006). Human mesenchymal stem cells exert potent antitumorigenic effects in a model of Kaposi’s sarcoma. J Exp Med.

[72] Cavarretta IT (2010). Adipose tissue–derived mesenchymal stem cells expressing prodrug-converting enzyme inhibit human prostate tumor growth. Mol Ther.

[73] Hassanzadeh A, Shamlou S, Yousefi N, Nikoo M, Verdi J (2022). Genetically-modified stem cell in regenerative medicine and cancer therapy; a new era. Curr Gene Ther..

[74] Kucerova L (2015). Tumor-driven molecular changes in human mesenchymal stromal cells. Cancer Microenviron.

[75] Djouad F, Plence P, Bony C, Tropel P, Apparailly F, Sany J, Noël D, Jorgensen C (2003). Immunosuppressive effect of mesenchymal stem cells favors tumor growth in allogeneic animals. Blood.

[76] Honkala A, Malhotra SV, Kummar S, Junttila MR (2022). Harnessing the predictive power of preclinical models for oncology drug development. Nat Rev Drug Discov..

[77] Ganguly K, Kimmelman AC (2023). Reprogramming of tissue metabolism during cancer metastasis. Trends in Cancer..

[78] Bressy C, Droby GN, Maldonado BD, Steuerwald N, Grdzelishvili VZ (2019). Cell cycle arrest in G2/M phase enhances replication of interferon-sensitive cytoplasmic RNA viruses via inhibition of antiviral gene expression. J Virol.

[79] Stewart JH, Ahmed M, Northrup SA, Willingham M, Lyles DS (2011). Vesicular stomatitis virus as a treatment for colorectal cancer. Cancer Gene Ther..

[80] Fani M, Zandi M, Rezayi M, Khodadad N, Langari H, Amiri I (2018). The role of microRNAs in the viral infections. Current Pharmaceutical Design..

[81] Pan W, Bodempudi V, Esfandyari T, Farassati F (2009). Utilizing ras signaling pathway to direct selective replication of herpes simplex Virus-1. PLoS ONE.

[82] Carew JS, Espitia CM, Zhao W, Mita MM, Mita AC, Nawrocki ST (2017). Oncolytic Reovirus inhibits angiogenesis through induction of CXCL10/IP-10 and abrogation of HIF activity in soft tissue sarcomas. Oncotarget.

[83] Zhang BO, Li YL, Zhao JL, Zhen O, Yu C, Yang BH, Yu XR (2018). Hypoxia-inducible factor-1 promotes cancer progression through activating AKT/Cyclin D1 signaling pathway in osteosarcoma. Biomed Pharmacother..

[84] Callegari E, Elamin BK, D’Abundo L, Falzoni S, Donvito G, Moshiri F (2013). Anti-tumor activity of a mir-199-dependent oncolytic adenovirus. PLoS ONE.

[85] Dix BR, O’Carroll SJ, Myers CJ, Edwards SJ, Braithwaite AW (2000). Efficient induction of cell death by Adenoviruses requires binding of E1B55k and P53. Cancer Res.

[86] Menotti L, Nicoletti G, Gatta V, Croci S, Landuzzi L, De Giovanni C (2009). Inhibition of Human Tumor Growth in Mice by an Oncolytic Herpes Simplex Virus Designed to Target Solely HER-2-Positive Cells. Proc Natl Acad Sci..

[87] Jing Y, Zaias J, Duncan R, Russell SJ, Merchan JR (2014). In vivo safety, Biodistribution and Antitumor Effects of uPAR Retargeted Oncolytic Measles Virus in Syngeneic Cancer Models. Gene Ther.

[88] Sakhawat A, Ma L, Muhammad T, Khan AA, Chen X, Huang Y (2019). A tumor targeting oncolytic adenovirus can improve therapeutic outcomes in chemotherapy resistant metastatic human breast carcinoma. Sci Rep..

[89] Ali S, Tahir M, Khan AA, Chen XC, Ling M, Huang Y (2019). Cisplatin synergistically enhances antitumor potency of conditionally replicating adenovirus via p53 dependent or independent pathways in human lung carcinoma. Int J Mol Sci..

[90] Sakhawat A, Liu Y, Ma L, Muhammad T, Wang S, Zhang L, Cong X, Huang Y (2017). Upregulation of Coxsackie adenovirus receptor sensitizes cisplatin-resistant lung cancer cells to CRAd-induced inhibition. J Cancer.

[91] Gao S, Yang X, Xu J, Qiu N, Zhai G (2021). Nanotechnology for boosting cancer immunotherapy and remodeling tumor microenvironment: the horizons in cancer treatment. ACS Nano..

